# Climate change, thermal anomalies, and the recent progression of dengue in Brazil

**DOI:** 10.1038/s41598-024-56044-y

**Published:** 2024-03-11

**Authors:** Christovam Barcellos, Vanderlei Matos, Raquel Martins Lana, Rachel Lowe

**Affiliations:** 1 Climate and Health Observatory, Institute of Health Information and Communication, Oswaldo Cruz Foundation (ICICT/Fiocruz), Avenida Brasil 4365, Manguinhos, Rio de Janeiro, RJ 21040-900 Brazil; 2 Barcelona Supercomputing Center (BSC), Barcelona, Spain; 3 Catalan Institution for Research and Advanced Studies (ICREA), Barcelona, Spain; 4 Centre on Climate Change and Planetary Health and Centre for Mathematical Modelling of Infectious Diseases, London School of Hygiene and Tropical Medicine, London, UK

**Keywords:** Climate change, Infectious diseases, Disease prevention, Public health

## Abstract

Dengue is rapidly expanding its transmission area across Brazil and much of South America. In this study, data-mining techniques were used to identify climatic and demographic indicators that could explain the recent (2014–2020) and simultaneous trends of expansion and exacerbation of the incidence in some regions of Brazil. The previous circulation of the virus (dengue incidence rates between 2007 and 2013), urbanization, and the occurrence of temperature anomalies for a prolonged period were the main factors that led to increased incidence of dengue in the central region of Brazil. Regions with high altitudes, which previously acted as a barrier for dengue transmission, became areas of high incidence rates. The algorithm that was developed during this study can be utilized to assess future climate scenarios and plan preventive actions.

## Introduction

The distribution of dengue and other arboviruses has been changing in recent decades with the expansion of transmission areas towards zones of higher latitude and altitude^1,2^. Part of the problem is due to the difficulties of controlling the vector^3z^, and its ability to adapt to the built environment^3^. Incomplete urbanization, that is, without promoting adequate infrastructure and services for the occupation of cities, is also an important socio-environmental determinant of dengue^4^. Although dengue occurs worldwide, the recent upward trend is more accelerated in Latin America and Asia, which has exposed an increasing amount of the world's population^2^. The rapid spread of Zika in tropical countries between 2013 and 2016 is an unequivocal demonstration of the presence of the *Aedes* vector in these parts of the world, as well as the ability of the virus to circulate through infected people, facilitated by air and ground transport^5,6^. Several studies have also shown the influence of increased temperature and precipitation on the occurrence of dengue outbreaks as reviewed by Damtew^7^, which may be related to global processes such as climate change.

In Brazil, a recent expansion of the dengue transmission area towards the south and center of the country has been observed^8,9^. Among the socio-environmental factors implicated is the occurrence of extreme weather events, such as droughts and floods^10^, and the expansion of the economic frontier towards the Amazon through the construction of roads and the degradation of pristine forest^11,12^. However, few studies have focused on medium and long-term trends that explicitly demonstrate the influence of long-term temperature increases on the expansion of the transmission area and the sustainability of transmission over decades. The lack of historical and large-scale data constrains empirical studies of long-term trends and the attribution of any trends to climate change. Although there is a clear upward trend in the average global surface temperature^13^, the distribution of heat in each region can vary strongly, depending on vegetation cover, the effect of ocean–atmosphere interactions such as El Niño Southern Oscillation (ENSO) and natural forcings such as volcanoes^14^. Thus, both the temperature and the incidence of dengue can suffer oscillations in space and time, which requires appropriate methods and approaches to disentangle climate and disease dynamics.

Temperature is a key environmental factor that regulates mosquito infestation and therefore dengue transmission. The reproduction of the *Aedes* mosquito is viable between 18 and 33 °C^15^, and the optimal range for maintaining arbovirus transmission is 21–30 °C^16^, a pattern that occurs in most of Brazil. However, in some high areas of the Central Plateau and the Southern region, average temperatures below 18 °C are observed, mainly during the winter. The increase in temperature above this value in winters in zones previously considered temperate can maintain the vector reproduction cycle and allow the sustainability of disease transmission throughout the year, not only the occurrence of outbreaks. This pattern change may occur in other regions of the world that are experiencing climate change, which may be more long-lasting and pose a risk of arbovirus transmission in new areas, such as southern Europe, North America, and Northeast Asia, among others^2,3^. Brazil, due to its climatic diversity and territorial extension (latitudes ranging from 1° North to 30° South), can be taken as an example of these climatic and demographic processes that are underway in the world. The CDC^17^ classifies the risk of dengue in countries according to three categories: "Frequent or continuous risk", which means that either frequent outbreaks occur or transmission is ongoing; "Sporadic or uncertain risk", which means that the risk varies and is unpredictable"; and "no evidence of risk". The distinction between these categories is fundamental for establishing appropriate strategies for vector and disease monitoring^18^, control and care, depending on the cases are sporadic and imported emerging and increasing, or endemic. There are multiple ways to model dengue dynamics and its climatic and environmental determinants. Depending on the research question, the outcome of interest might be incidence rates interannual variability, seasonality, intensity, the occurrence of outbreaks, etc. Climatic variables might include rainfall, humidity, temperature, or their interactions, which can be synthesized as averages, medians, frequency, and probability of occurrence of extreme events. To address this variable selection challenge, data mining techniques are a useful tool to identify the most suitable variables to describe the disease transmission process. To date, this kind of technique has been scarcely employed in the analysis of complex problems, such as the impact of climate variability and climate change on the transmission of arboviruses^19,20^. In this context, this study evaluates the climatic and demographic factors that contribute to the spread of dengue and its establishment as an endemic disease with persistent transmission. We use data mining techniques to evaluate association between thermal anomalies, demographic factors and changing dengue incidence patterns over a 21-year period (2000–2020) across the microregions of Brazil. Recent reports made public by the press and epidemiological bulletins claim that dengue was showing wider spatial dispersion, a record numbers of cases, and reaching new areas that were previously transmission-free. This study was designed to respond to these hypotheses, and as an approach to compliment long-term monitoring of climate-sensitive diseases.

## Material and methods

### Study area

**Figure 1:**
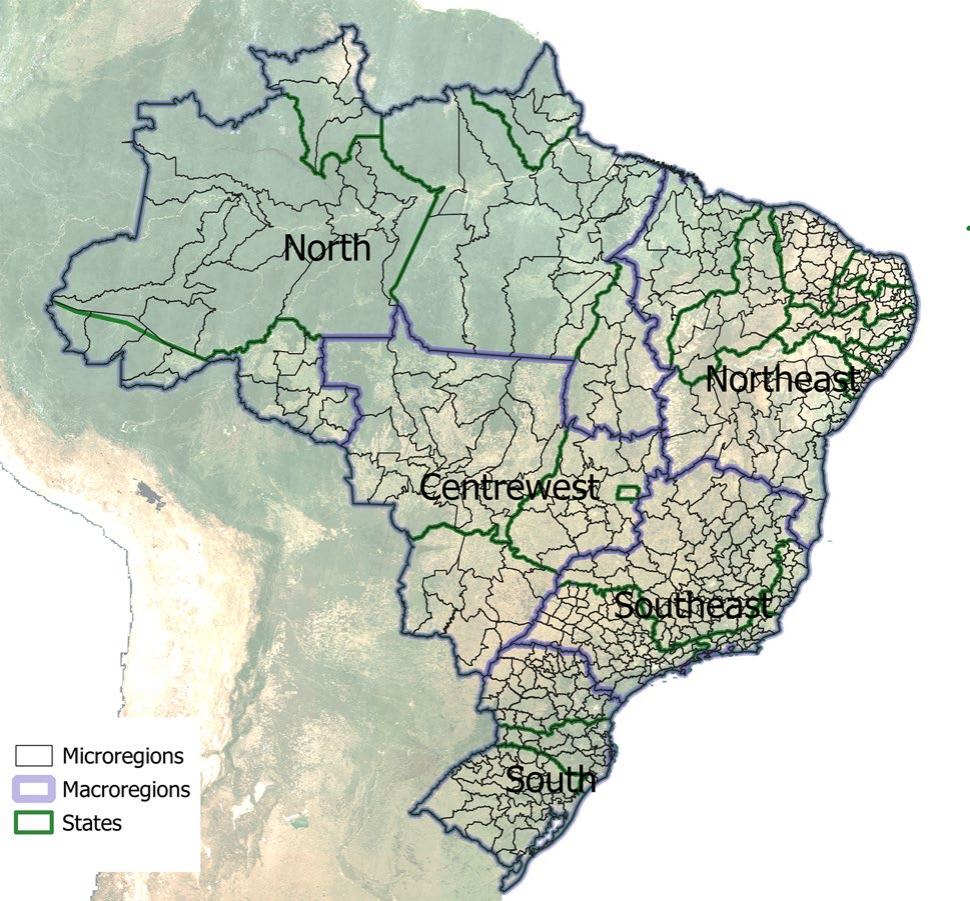
Boundary limits of Brazilian territories: 5 regions, 26 states and 1 federal district, and 553 microregions.

Dengue notification, population, and climate data were collected and aggregated according to the 553 microregions (MRG) of the country (see supplementary files). The microregions represent aggregations of the 5568 municipalities in the country, according to the homogeneity of socioeconomic characteristics and the existence of a network of small to medium-sized cities^21^. Figure 1 shows the boundaries of microregions, states and regions in Brazil. By constructing indicators in larger and more uniform areas than the administrative divisions, the statistical instability of spatial units with a small population is reduced, as well as the effect of mixing heterogeneous municipalities^22^.

### Data

Dengue case data, confirmed by clinical or laboratory tests, were obtained from SINAN between 2000 and 2020 and aggregated over 7-year periods (2000–2006, 2007–2013, and 2014–2020). These longer intervals allow the evaluation of long-term trends without the interference of specific outbreaks, and low incidence years following large epidemics^23^. The division of the time series into three periods sought to separate the phases of the space–time progression of dengue in Brazil, which is marked by periods of more rapid expansion and the occurrence of major epidemics (see Fig. S1 in the supplementary material for details).

Dengue incidence rates (DIR) were calculated for each microregion (see Table 1 in supplementary material) summing the number of notified cases and dividing by the estimated population per 100,000 for the 3 time periods. Dengue transmission was classified into three bands that are used by the Brazilian unified health system (SUS) as references for identifying outbreaks and issuing alerts: no incidence (zero rate), low-to-medium incidence (rate between 1 and 300 cases per 100,000 inhabitants); and high incidence (rate greater than 300 cases per 100,000 inhabitants).

The total population of each period was obtained from the demographic census 2000 and 2023 and estimates for the intermediate years were carried out by the Ministry of Health (DATASUS). The proportion of residents living in urban areas and the population growth rate in each period was also calculated.

**Figure 2:**
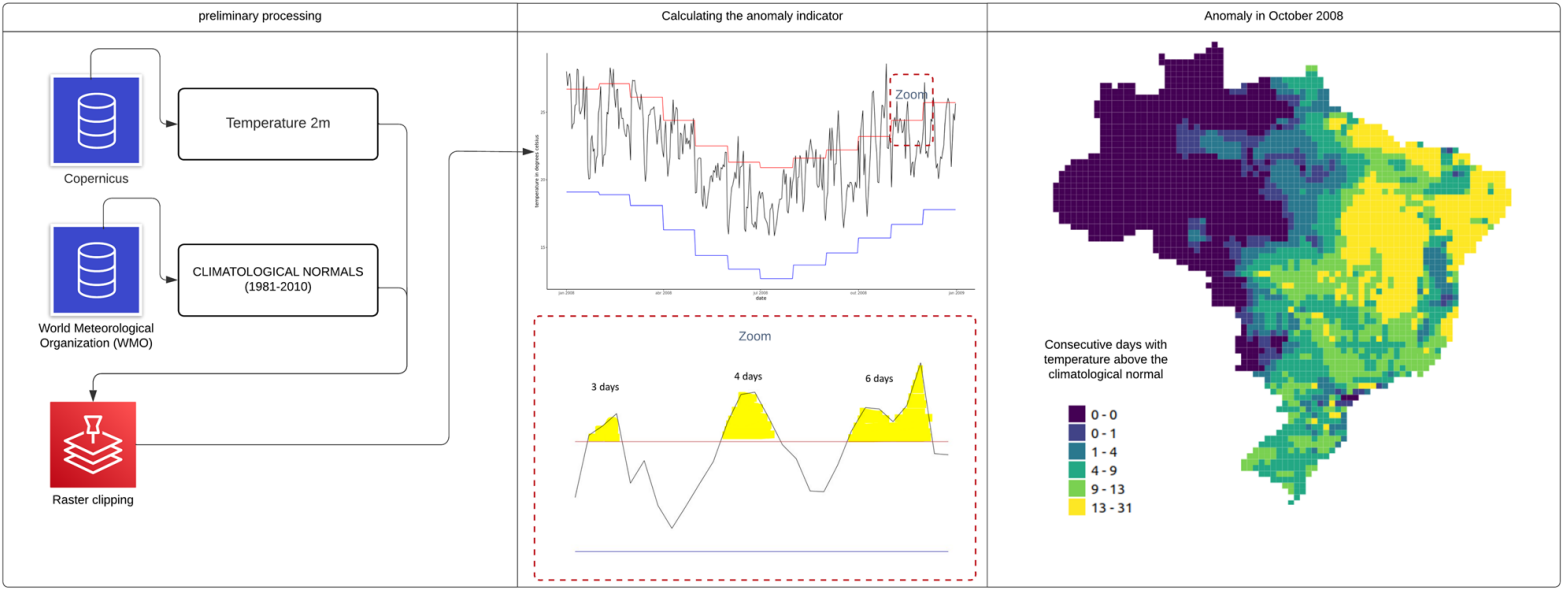
Approach for calculating the thermal anomalies climate indicator for dengue.

The occurrence of temperature anomalies was evaluated according to an algorithm that calculates the difference between the observed temperature and the mean of the historical series, considering its seasonality. Figure 2 outlines the treatment of the variables for the production of the thermal anomaly indicator. We consider a climatic anomaly when the temperature of a place exceeds the maximum temperature of the reference climatological normal (1981–2010).

Figure 2 illustrates the steps used to calculate anomalies for October 2008: First, a pre-processing the different raster files containing daily temperature were merged into a unique file. The daily temperature (obtained from the Copernicus service catalog in the ERA Land5 system)^24^ and the raster files with normal climatological reference data (obtained from the World Meteorological Organization available at https://www.ncei.noaa.gov/pub/data/normals/WMO/1981-2010/RA-III/) were geoprocessed by clipping each file by a grid layer spacing 0.5° latitude and longitude. Then, in the indicator calculation stage, the value of the maximum daily temperature was related to each cell in the grid and the frequency that the maximum daily temperature exceeds its reference value of the climatological normal was recorded. In this way, the number of days in which the observed temperature exceeds the maximum of the normal climate of the place is found, and after a few cycles of repetition of the same process, we obtain the changes in climate patterns for each cell of the grid, at the end the result was aggregated by the average for the microregions in which they are contained using QGis software (version 3.30). GIS was also used to produce visualizations of the calculated thermal and dengue incidence.

The number of days with maximum and minimum temperature anomalies was calculated for the three periods, during the whole years and for the summer months (January–March) in order to identify whether summer anomalies influence higher dengue incidence and whether low temperature anomalies can reduce incidence. In addition, altitude values were collected from the average altitude of the center of the municipalities, since this factor has been pointed out as limiting dengue transmission in other studies^25^.

Population increase during the last period (2014–2020) was calculated in order to assess the recent changes in demography. Rapid population growth in some areas can cause difficulties in accessing urban services such as water supply, waste collection and adequate housing, as well as the qualification of health services itself that can have difficulties in the diagnose and notification of dengue cases. Population density (in inhabitants per km^2^) and the proportion of urban population, were also calculated since it affects the ecology of mosquitos and urban infrastructure. Data sources and mean values are shown in supplementary material (Table S1).

### Statistical analysis

The epidemiological, climatic and demographic indicators aggregated by microregion were organized in a single dataset and explored using univariate and multivariate statistical analysis. Multiple linear regression analysis having as the outcome variable (dependent) the dengue incidence rate (DIR) from 2014 to 2020 was employed to verify the most important variables and their collinearity, by applying the stepward method with the removal of non-significant variables in the model (α > 0.05).

The second phase consisted of analysing a selected number of variables using data mining techniques. The main advantage of this approach is the discovery of patterns using large data sets with many different possible explanatory variables. Regression trees were generated using the CHAID method to identify the main indicators of climate and demography that may explain the spatial patterns of dengue incidence. The regression tree seeks to classify groups of microregions based on independent variables, and along this tree establishes cutoff points of these variables—which are not necessarily normally distributed—that better distinguish regions of greater or lesser value from the dependent variable^26^. The data mining approach effectively identifies complex interactions between contextual variables without a priori specification of interaction terms.

## Results

**Figure 3:**
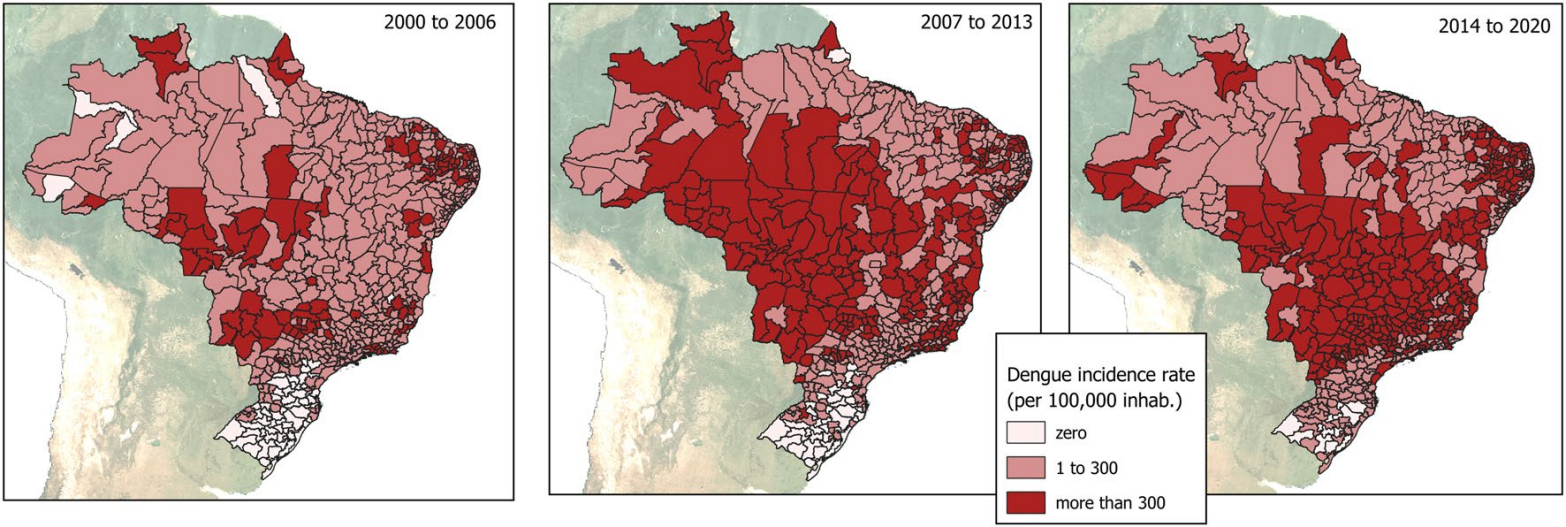
Dengue incidence rates in microregions of Brazil between 2000 to 2006, 2007 to 2013 and 2014 to 2020 per 100,000 inhabitants.

The following maps (Fig. 3) show the evolution of dengue distribution in the study periods.

Both the spread of the disease throughout the territory, as well as the worsening of dengue in some regions of the country is observed over time. The central portion of the country (Centre West region, with a predominant savanna like biome), for example, has reported dengue cases since the beginning of the historical series, and the highest incidence rates of the country since 2014. The North region (Amazon biome) has become an area of permanent transmission, even with low incidence rates and the emergence of some microregions of high risk of transmission, located at the edges of recent occupation of the region. There is an alarming number of microregions in the more recent period (2014–2020) with very high rates (DIR > 300) in an area that covers the western part of the Southeastern region, as well as the south of the Centre West region, which were areas of low incidence in previous periods. This area largely coincides with the Paraná River basin.

Table 1 shows the categories of dengue incidence and the category transition from 2000 to 2006 to 2007 to 2013. Table 2 shows the changes between 2007 to 2013 and 2014 to 2020.

**Table 1:** Number of microregions classifications according to dengue incidence in the period from 2007 to 2013 in relation to the previous period from 2000 to 2006.

		Dengue Incidence rate 2007–2013 (per 100,000 inhab.)			Total
		0	1 to 300	More than 300	
Dengue incidence rate 2000–2006 (per 100,000 inhab.)	0	10	13	0	23
	1–300	3	279	138	420
	More than 300	0	25	85	110
Total		13	317	223	553

**Table 2:** Number of microregions per classification according to dengue incidence in the period from 2014 to 2020 in relation to the previous period from 2007 to 2013.

		Dengue incidence rate 2014–2020 (per 100,000 inhab.)			Total
		0	1–300	More than 300	
Dengue Incidence rate 2007 to 2013 (per 100,000 inhab.)	0	2	11	0	13
	1–300	1	198	118	317
	More than 300	0	49	174	223
Total		3	258	292	553

There was a small but relevant change in the classification of microregions in relation to dengue incidence. From 2007 to 2013, a total of 13 (2.3%) microregions which had not reported cases (zero rates) transitioned to a low incidence range of 1–300 cases per 100,000 inhabitants.). Only ten (1.8%) microregions remained without cases. In the same period, 138 (24.9%) microregions jumped from the low range to the highest (epidemic) level of dengue incidence.

In the last period (2014–2020), only two (0.3%) microregions remained without cases. Eleven microregions with no cases between 2007 and 2013 moved into the low incidence category (1.9%). Of the 317 microregions in the low range, 118 (21.3%) jumped to the epidemic level, and only one microregion returned to zero incidence values. Of the 223 microregions in the high incidence category (more than 300 cases per 100,000 inhabitants), only 49 (8.9%) returned to low values (1–300 cases per 100,000 inhabitants).

What emerges from Table 2 and the maps (Fig. 3) is that the process of expansion of the dengue transmission area appears to be irreversible. Once the virus and the vector are introduced, it is unlikely to return to a zero-transmission scenario. This situation is even more serious in microregions with higher incidence rates (more than 300 cases per 100,000 inhabitants), in which about 77% of the microregions tended to remain with rates considered epidemic from 2007 to 2013 and 2014 to 2020. Currently, 52% of the country's microregions (292/553) are in the dengue epidemic range, and 46% (258/553) are in the medium to low incidence range, in which "sporadic or uncertain risk" can be observed^17^. During the last period, only three microregions remain with no dengue cases, all located in the extreme South of the country.

**Figure 4:**
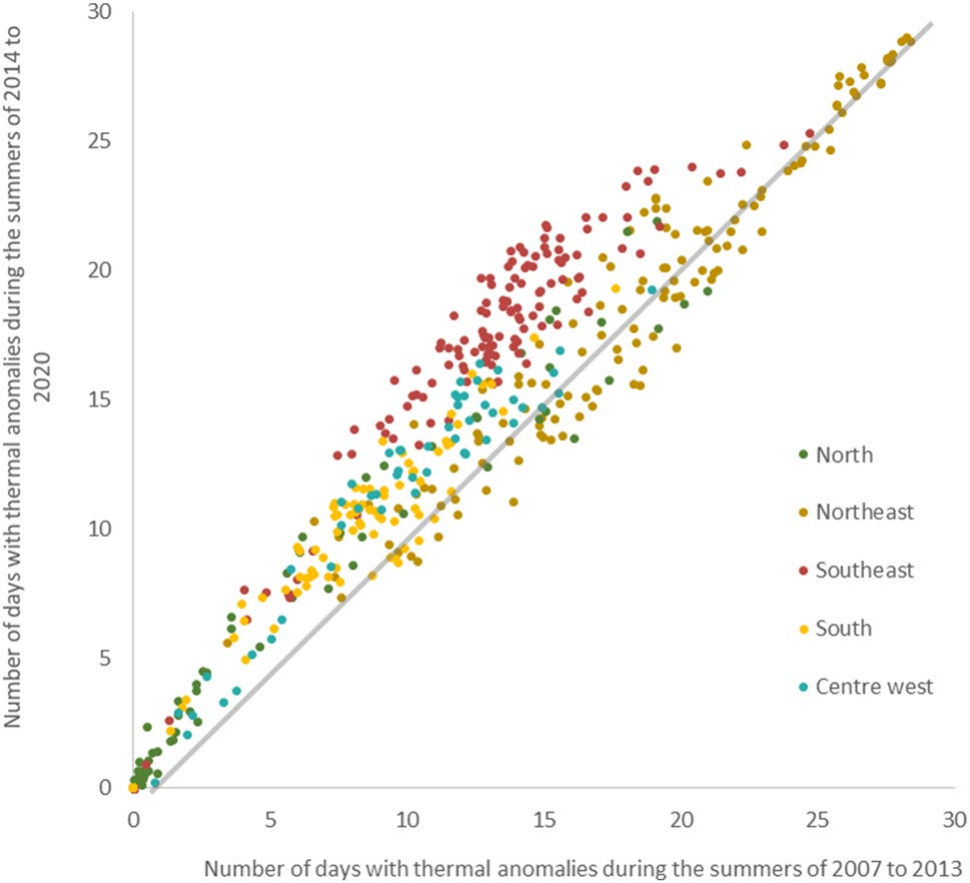
Relationship between temperature anomalies, in days per month of the summer period, in microregions of the Brazilian regions between the periods of 2007 to 2013 and 2014 to 2020.

Figure 4 compares the occurrence of temperature anomalies in microregions of the five Brazilian regions between 2007 to 2013 and 2014 to 2020.

**Figure 5:**
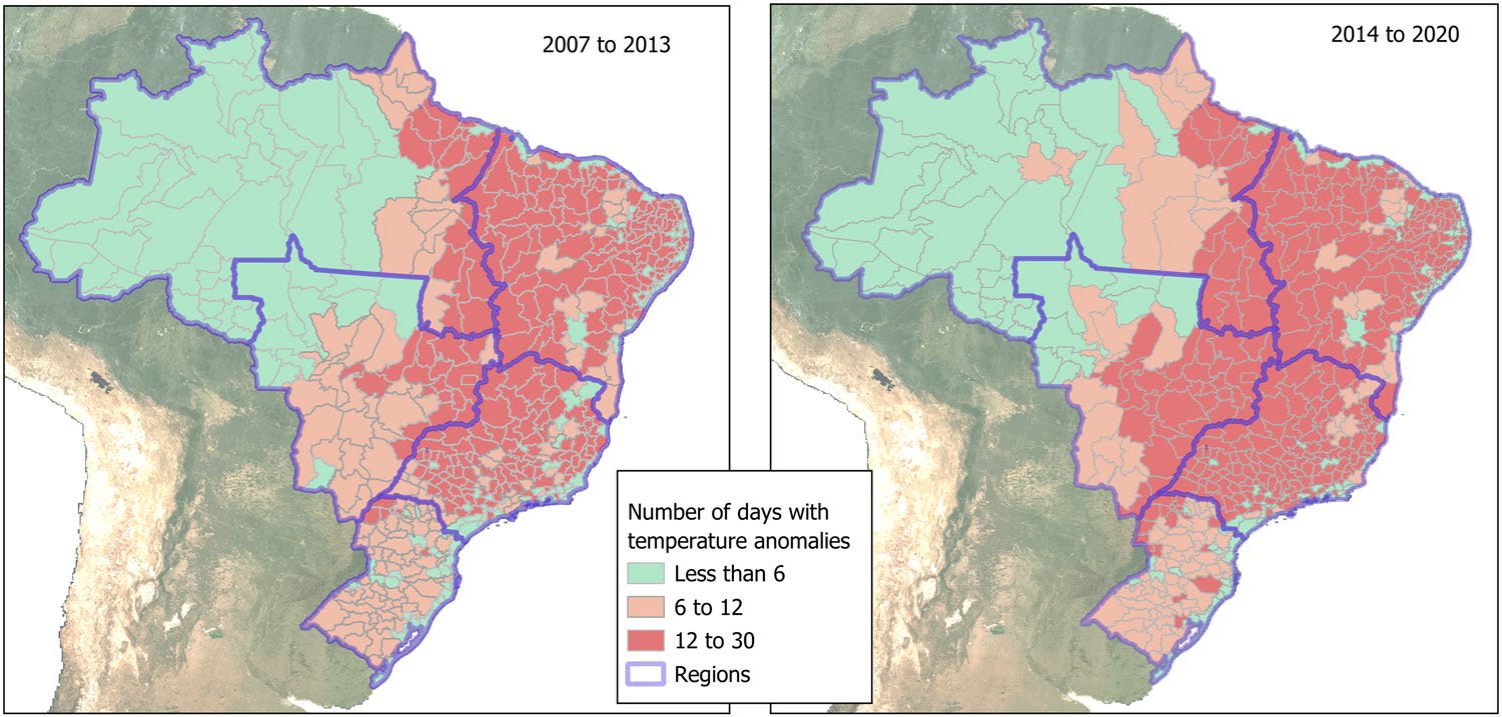
Number of days per month with maximum temperature anomalies during the summers from 2007 to 2013 and from 2014 to 2020.

The Southeast region, which had already been presenting recurrent thermal anomalies, has had the largest increase in the occurrence of 10–24 days of temperature anomalies per month. The South region, which had a low frequency of anomalies, began to have up to 10 days of temperature anomalies per month in recent years. The Northeast region presents the highest frequency of temperature anomalies, with at least 5 days of the month with thermal anomalies, however, without marked changes. The North region has not experienced anomalously high temperatures throughout the period. Figure 5 shows the frequency of warm days, measured according to the algorithm for calculating maximum thermal anomalies.

Between 2007 and 2013, most of the central regions of Brazil suffered anonymously warm temperatures, more frequent in the Northeast and Southeast regions and less frequent in the South and Centre West. In the following period, from 2014 to 2020, anomalies increased throughout the country, with greater frequency in the South and Centre West. Most coastal areas and the Amazon had a low frequency of temperature anomalies throughout the period of 2007–2020.

Table 3 shows the results of multiple linear regression analysis of dengue incidence rate from 2014 to 2020 as response variable and different combinations of demographic and climatic indicators as the explanatory variables.

**Table 3:** Regression coefficients (crude and standardized) selected in the multiple regression analysis for dengue incidence rate in the period from 2014 to 2020: incidence rate in the previous period, between 2007 and 2013; altitude; and number of maximum temperature anomalies during summers between 2014 and 2020.

	Unstandardized Coefficients		Standardized Coefficients	t	α
	Value	Std. Error			
(Constant)	30.72	48.72		0.63	0.52
Dengue incidence rate (per 100,000 inhab.) between 2007 and 2013	0.56	0.06	0.38	10.06	< 0.001
Altitude (m)	0.51	0.08	0.25	6.68	< 0.001
Number of days with thermal anomalies during summers (2014–2020)	15.84	3.67	0.16	4.31	< 0.001

**Figure 6:**
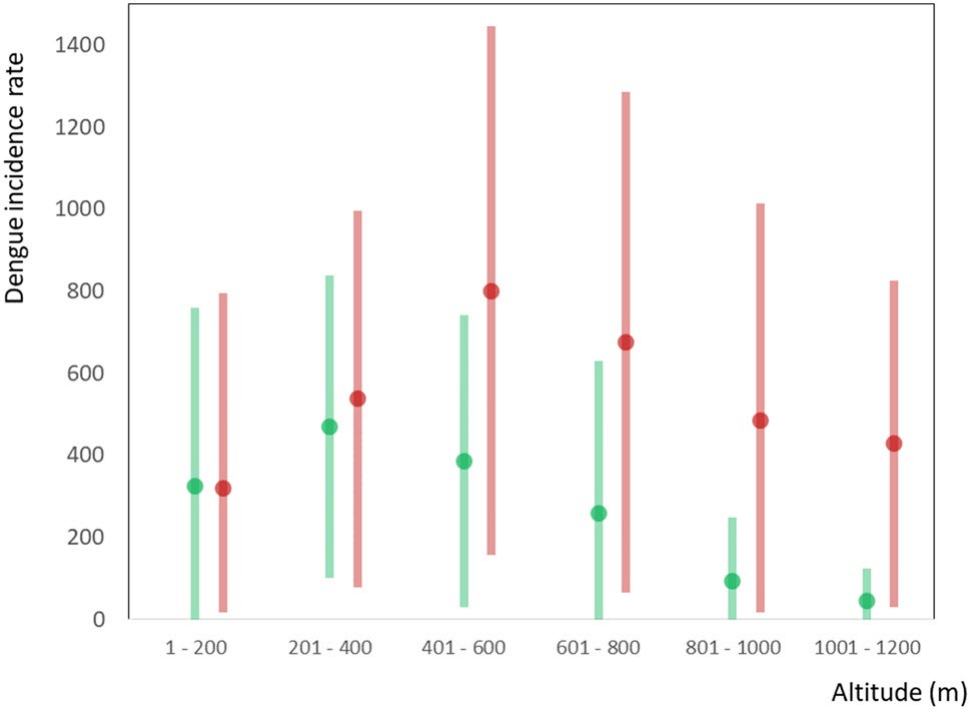
Variation of mean and standard deviation dengue incidence rate (cases per 100,000 inhabitants) in two periods:2007–2013 in green and 2014–2020 in red, and the altitude ranges of the microregions (in meters).

The most important variables, according to the model, were the incidence rate in the previous period (2007–2013), the average altitude and the occurrence of maximum temperature anomalies in the same period (2014–2020). During this period, the expansion of areas with high DIR in microregions located in the Central Plateau of the country was also observed, reaching higher altitudes of the South and Southeastern regions, which is demonstrated by the significant weight of altitude variable in the final regression model. For every 100 m, there is an increase in the incidence of about 50 dengue cases per 100,000 inhabitants. The regression analysis using all the variables collected for this study, is presented in the annex. Figure 6 shows a shift in the pattern of dengue incidence along altitude gradients in Brazil.

**Figure 7:**
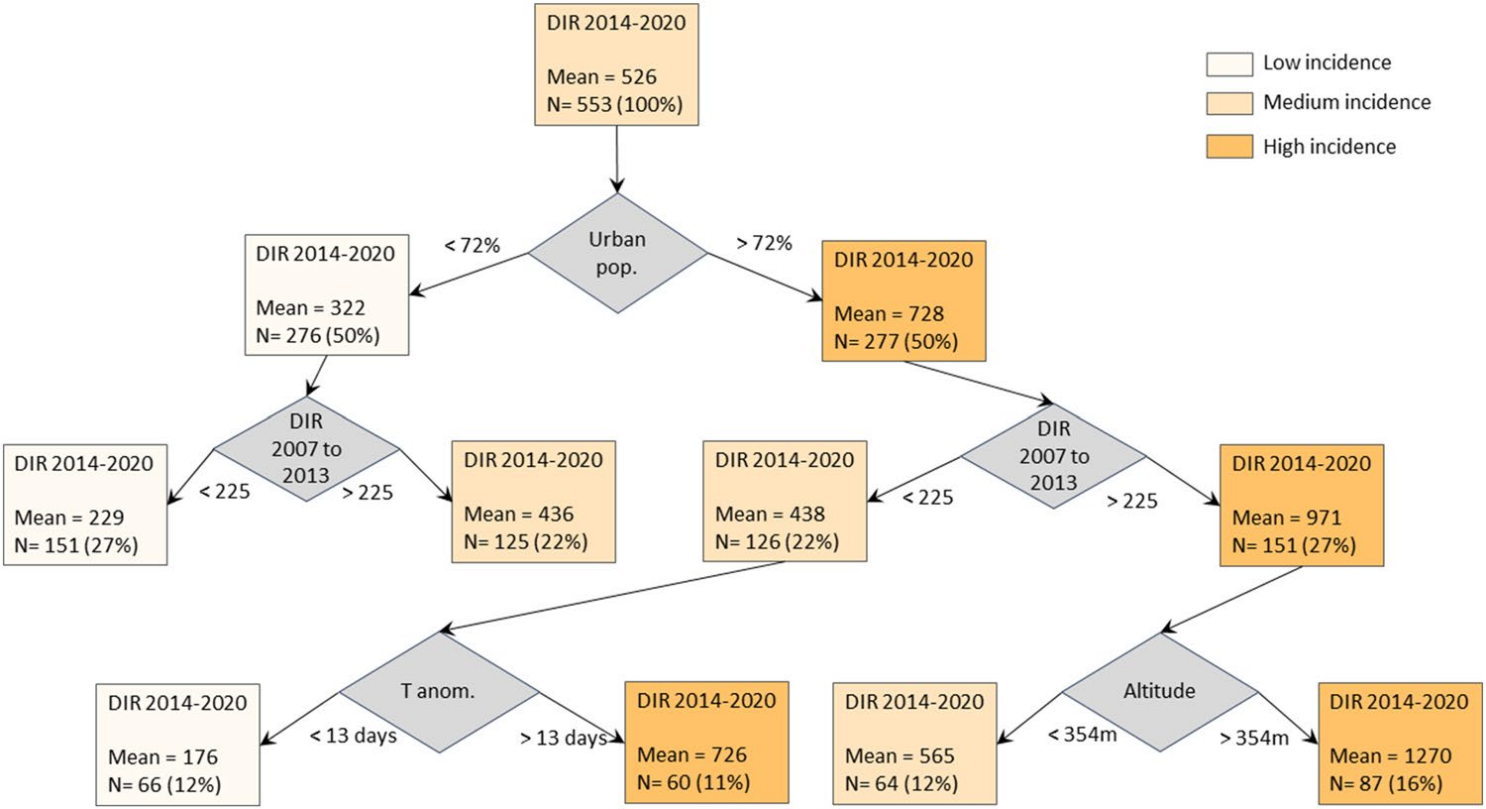
Decision tree explaining the dengue incidence rate (DIR) in the period from 2014 to 2020, highlighting the branches established by the variables: proportion of people living in urban areas; dengue incidence rate in the previous period, from 2007 to 2013; the number of maximum temperature anomalies in summer in the same period (2014–2020); and altitude in meters. Mean of DIR and the number of microregions is shown for each tree branch.

In the first period (dots and line in green), there is a sharp decrease in DIR with increasing altitude. In line with other studies^25^, the almost absence of areas with cases above 1000 m of altitude is observed. In the most recent period (2014–2020), the incidence of dengue even tends to increase with altitude up to the range of 600 m, falling towards higher altitudes. This new pattern of dengue distribution in Brazil may reveal the recent expansion of transmission areas towards the plateaus and mountains. The effect of these temperature anomalies and other demographic factors can be seen in the regression tree (Fig. 7).

Microregions with greater urbanization (urban population greater than 72% of the total) was positively associated with dengue incidence rate (DIR). Of these more urbanized microregions, there was an increase in the dengue incidence rate in those microregions that already had high incidence rates (more than 225 cases per 100,000 inhabitants) in the previous period (2007–2013), while the incidence rate in microregions with lower rates further declines in the more recent period. Among the microregions with low incidence rates (less than 225 cases per 100,000 inhabitants), there was an increase in the incidence in microregions that were subjected to positive thermal anomalies for a period longer than 13 days a month during the summer. On the other hand, among the microregions with the highest incidence rates (more than 225 cases per 100,000 inhabitants), there was an even more significant increase in microregions of higher altitudes (more than 354 m).

## Discussion and conclusion

The groups of microregions that presented the highest dengue incidence rates in the more recent years were those with a previous base level of dengue transmission and a high degree of urbanization. In addition, climatic conditions, such as the occurrence of positive thermal anomalies, have aggravated dengue incidence rates, even in areas with historically low incidence rates. In recent decades, the expansion of dengue fever towards the Central Plateau has been observed in the western areas of the South and Centre West regions, as well as inner regions of Northeast region, along the Borborema Plateau. Higher altitude areas, previously considered a limiting factor in the transmission of dengue, now represents a geographical zone susceptible to the expansion of the area of transmission of dengue and other arboviruses. In this case, the effect of the first epidemic waves of the disease, after the proliferation of *Aedes* vector and the introduction of the virus and when encountering a large part of the susceptible population, should be considered^27^. The corridor that is now consolidating in the southern hinterland of Brazil, marked here by the occurrence of thermal anomalies and increase in the incidence of dengue, extends into northern Argentina, Uruguay and Paraguay^28^.

The period of 2–3 weeks, which approaches the cutoff points of 11–13 days observed for the indicator of thermal anomalies in this study, may indicate that there is a minimum duration of warm periods capable of sustaining and even accelerating the reproduction of the vector^9^. According to the results of this study, the frequency of thermal anomalies during the summer is the most explanatory climatic indicator of the increase in dengue incidence rates in the long term. A recent study carried out in Argentina showed that temperature anomalies played a more important role in the spread of the epidemic than the increase in average temperature or total rainfall^28^. Other indicators were tested, such as the occurrence of anomalies of maximum temperatures throughout the year and the anomalies of minimum temperatures in the summer and throughout the year, without significant results, both in the linear regression model and in the regression tree. This indicator can be easily incorporated into epidemic warning systems since it is easily accessible and interpreted. The methodology adopted allowed the identification of cutoff points (the duration of anomalies, on days of the month with temperatures above the historical level) that can serve as an input for early warning and response systems^29^. The Brazilian Climate and Health Observatory will implement this and other indicators in its data platform to improve surveillance of vector-borne diseases at the national and regional levels.

Areas of Brazil with higher rates in the last period (2014–2020) were more urbanized, with incidence rates high enough for sustained transmission throughout the year, higher altitudes or areas subjected to high temperatures for a prolonged period of time. This points to the medium and long-term effects of global warming and its possible effects on regional climates.

Among the limitations of this study are issues inherent to the scale and the temporal and spatial aggregations of climatic and epidemiological variables. The variables and parameters identified in this space and time study frame may not be suitable for a single city, that is, dengue outbreaks are not always a result of local temperature anomalies. This study shows that the geographic position and other demographic characteristics of cities potentiate the effect of thermal anomalies. However, the variables adopted in this study, such as the degree of urbanization, altitude, dengue incidence in the previous period and the occurrence of temperature anomalies, may be good predictors of the expansion of the transmission areas in the near future. In this study, the number of days with maximum temperature above the historical level was used to distinguish climate change trends, as suggested by Lins et al.^30^. New climatological indicators should be sought, such as the maximum annual value of the minimum daily temperature, the minimum annual value of the maximum daily temperature, and daily temperature variability, always in a manner consistent with the biological parameters that regulate the intrinsic and extrinsic cycle of the virus and the reproduction speed of the *Aedes* mosquito, which are strongly influenced by temperature^31^. Not only that, but temperatures above normal influence human behavior and can promote exposure to the vector in open places, houses without air conditioning and the change of clothing in periods of extreme heat^32^.

Whatever the mechanisms that explain the higher incidence in areas that have suffered temperature anomalies, this work demonstrates the possible medium-term effects of thermal anomalies on the increase of dengue transmission, as well as its expansion towards previously unaffected areas of higher altitude and latitude, which have been undergoing warming and changes in the rainfall regime. With the development of seasonal climate forecasting systems, the patterns discovered in this study can serve as early indicators for generating alerts of dengue epidemics to allow for anticipatory actions.

### Supplementary Information

Supplementary Information.

## Data Availability

The datasets generated during the current study were collected from open and free data platforms and are available on Zenodo repository under https://doi.org/10.5281/zenodo.10404906. The algorithm code developed to generate indicators of climate anomalies is available at https://doi.org/10.5281/zenodo.10592772 and https://github.com/vanderpascoal/ClimateCorr.
