# Author Correction: Climate change, thermal anomalies, and the recent progression of dengue in Brazil

**DOI:** 10.1038/s41598-024-58202-8

**Published:** 2024-03-28

**Authors:** Christovam Barcellos, Vanderlei Matos, Raquel Martins Lana, Rachel Lowe

**Affiliations:** 1 Climate and Health Observatory, Institute of Health Information and Communication, Oswaldo Cruz Foundation (ICICT/Fiocruz), Avenida Brasil 4365, Manguinhos, Rio de Janeiro, RJ 21040‑900 Brazil; 2 Barcelona Supercomputing Center (BSC), Barcelona, Spain; 3 Catalan Institution for Research and Advanced Studies (ICREA), Barcelona, Spain; 4 Centre on Climate Change and Planetary Health and Centre for Mathematical Modelling of Infectious Diseases, London School of Hygiene and Tropical Medicine, London, UK

Correction to: *Scientific Reports*, 10.1038/s41598-024-56044-y, published online 11 March 2024

The original version of this Article contained an error in Figure 1 label, where

**Figure 1:**
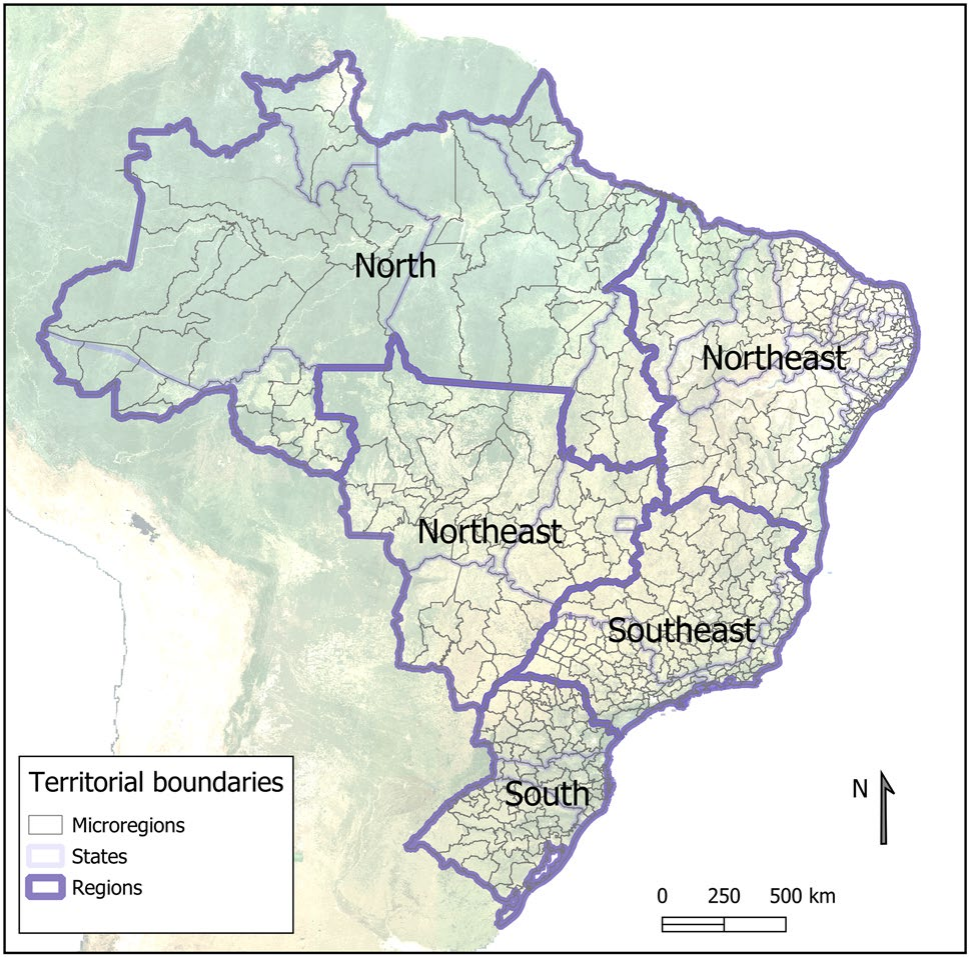
Boundary limits of Brazilian territories: 5 regions, 26 states and 1 federal district, and 553 microregions.

“Northeast”

now reads:

“Centrewest”

The original Figure 1 and accompanying legend appear below.

The original Article has been corrected.

