# Supplementary material for: Climate change, thermal anomalies, and the recent progression of dengue in Brazil
Source: Sci Rep. 2024 Mar 11;14:5948. doi: 10.1038/s41598-024-56044-y (PMC10928122; doi:10.1038/s41598-024-56044-y)
Supplement: Supplementary file 1 — Supplementary Information. [file 41598_2024_56044_MOESM1_ESM.docx]

**Climate change and thermal anomalies explain the recent progression of dengue in Brazil**

Supplementary information


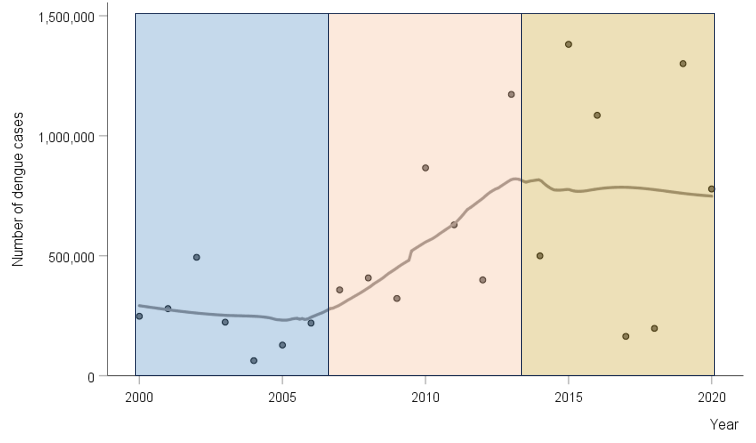


Figure S1: Number of dengue cases reported from 2000 to 2020. Three periods were discerned from the figure according to the slope of the interpolation line and the dispersion of the points along this line (occurrence of major epidemics).

The proposed periodisation (a time series divided into 7-year periods) sought to separate the stages of the (spatio-temporal) spread of the disease in Brazil. The first period from 2000 to 2006 is characterised by a permanent dengue transmission along the coastal regions of the country, mainly in large and medium-sized cities with warm climates. In the following period (2007 to 2013) transmission spread towards the south and the countryside, also increasing the overall incidence of the disease. During the last study period (2014 to 2020), the occurrence of major epidemics was observed, shown by the wide annual variation in the number of cases, in parallel to a new expansion cycle of the disease towards the interior and higher altitude areas of Brazil. In general, a year with high incidence is followed by one or two years of low incidence. This pattern of oscillation is a consequence of the immunity acquired by infected populations during previous outbreaks. Peaks in incidence may be also due to the entry of a new dengue serotype (DENV3). There are currently four types of dengue circulating in Brazil. In order to avoid interference from immunological factors and problems with the passive surveillance notification system, affected by the Covid-19 pandemic, longer time periods were defined.

Table S1: Description of variables used in this study. Data sources, indicator calculations, mean and range of values.

| **Variable code** | **Variable description** | **Information source** | **Mean** | **Range** |
| --- | --- | --- | --- | --- |
| nam_micro | Microregion name | IBGE, demographic census 2023 | - | - |
| cod_micro | Microregion code | IBGE, demographic census 2023 | - | - |
| region | Region name | IBGE, demographic census 2023 | - | - |
| cod_uf | State code | IBGE, demographic census 2023 | - | - |
| altitd | Mean altitude (m) | IBGE, demographic census 2023 | 384 | 2 to 1,405 |
| area | Total area (km^2^) | IBGE, demographic census 2023 | 15,431 | 276 to 336,242 |
| pop(2000 to 2020) | Total population | IBGE, interpolated from demographic census 2000 and 2023 | 382,254 | 20,512 to 15,132,944 |
| density | Population density | population / area | 113 | 0.3 to 6428 |
| pop_incrs | Population increase 2014 to 2020 (%) | (pop2020 - pop2014)*100/ (pop2020*6) | 1.7 | -4.3 to 15.8 |
| p_urb | Proportion of urban population (%) | popurb2020 *100/pop2020 | 71.9 | 23.62 to 99.93 |
| n_den_00_06 | Number of dengue cases 2000 to 2006 | SINAN, 2022 | 4,227 | 0 to 329,104 |
| n_den_07_13 | Number of dengue cases 2007 to 2013 | SINAN, 2022 | 5,725 | 0 to 458,890 |
| n_den_14_20 | Number of dengue cases 2014 to 2020 | SINAN, 2022 | 1,766 | 0 to 90,157 |
| dir_2000_06 | Dengue incidence rate 2000 to 2006 per 100,000 inhab. | (n_den_00_06 * 100000 / pop2003)/ 7 | 177 | 0 to 1,032 |
| dir_2007_13 | Dengue incidence rate 2007 to 2013 per 100,000 inhab. | (n_den_07_13 * 100000 / pop2010)/ 7 | 334 | 0 to 3,769 |
| dir_2014_20 | Dengue incidence rate 2014 to 2020 per 100,000 inhab. | (n_den_14_20 * 100000 / pop2017)/ 7 | 526 | 0 to 5,140 |
| max_month | Month of maximum dengue incidence | Jan = 1, feb= 2, mar= 3... | 3.5 | 0 to 12 |
| water_interm | Water intermittence -Frequency of declarations of interruptions in the water supply (%) | National Sanitation Information System (SNIS, 2020). | 34.7 | 0 to 100 |
| An_max_2007_13 | Thermal maximum anomalies 2007 to 2013 | Mean number of days with maximum temperature anomalies 2007 to 2013 | 12.2 | 0 to 29.7 |
| An_max_2014_20 | Thermal maximum anomalies 2014 to 2020 | Mean number of days with maximum temperature anomalies 2014 to 2020 | 7.1 | 0 to 26.2 |
| An_max_ver_2007_13 | Thermal maximum anomalies 2007 to 2013 during the summers | Mean number of days with maximum temperature anomalies 2007 to 2013 during the summers | 10.5 | 0 to 28.4 |
| An_max_ver_2014_20 | Thermal maximum anomalies 2014 to 2020 during the summers | Mean number of days with maximum temperature anomalies 2014 to 2020 during the summers | 12.1 | 0 to 29.0 |
| An_min_2007_13 | Thermal minimum anomalies 2007 to 2013 | Mean number of days with minimum temperature anomalies 2007 to 2013 | 4.4 | 0 to 29.9 |
| An_min_2014_20 | Thermal minimum anomalies 2014 to 2020 | Mean number of days with minimum temperature anomalies 2014 to 2020 | 3.7 | 0 to 29.5 |
| An_min_ver_2007_13 | Thermal minimum anomalies 2007 to 2013 during the summers | Mean number of days with minimum temperature anomalies 2007 to 2013 during the summers | 4.4 | 0 to 29.8 |
| An_min_ver_2014_20 | Thermal minimum anomalies 2014 to 2020 during the summers | Mean number of days with minimum temperature anomalies 2014 to 2020 during the summers | 3.7 | 0 to 29.4 |

Table S2: Linear regression using dengue incidence rate (per 100,000 inhab.) between 2014 and 2020 as dependent variable and climatic and sociodemographic variables as independent variables.

|  | | Unstandardized Coefficients | | | Standardized Coefficients | t | | Sig. |  |
| --- | --- | --- | --- | --- | --- | --- | --- | --- | --- |
|  |  | B | | Error | Beta |  |  |  |  |
| (Constant) | -742,4 | | 103,7 | |  | | -7,15 | ,00 |  |
| Altitude (Altitd) | ,35 | | ,08 | | ,177 | | 4,27 | ,00 |  |
| Population density | -,137 | | ,061 | | -,085 | | -2,22 | ,02 |  |
| Population increase (2014 to 2020) | -1,181 | | 11,86 | | -,004 | | -,09 | ,92 |  |
| Proportion of inhabitants living in urban areas | 11,527 | | 1,36 | | ,335 | | 8,43 | ,00 |  |
| Dengue incidence rate (per 100,000 inhab.) between 2007 and 2013 (tx2007_13) | ,431 | | ,05 | | ,295 | | 7,58 | ,00 |  |
| Intermittent water supply (%) | 129,303 | | 89,14 | | ,054 | | 1,45 | ,14 |  |
| An_max_2014_20 | 21,540 | | 6,07 | | ,222 | | 3,54 | ,00 |  |
| An_max_ver_2014_20 | -3,236 | | 4,71 | | -,046 | | -,68 | ,49 |  |
| An_min_2014_20 | 4,489 | | 3,86 | | ,045 | | 1,16 | ,24 |  |
| Dependent variable: Dengue incidence rate (per 100,000 inhab.) between 2014 and 2020 (tx2014_20) | | | | | | | | | |
